# The rare holley antibody associated with a severe hemolytic transfusion reaction: the importance of this antibody identification to find a compatible blood unit

**DOI:** 10.31744/einstein_journal/2020RC4582

**Published:** 2019-09-11

**Authors:** Leandro Dinalli Santos, Carolina Bonet Bub, Maria Giselda Aravechia, Eduardo Peres Bastos, Jose Mauro Kutner, Lilian Castilho

**Affiliations:** 1 Hospital Israelita Albert Einstein, São Paulo, SP, Brazil.; 2 Universidade Estadual de Campinas, Campinas, SP, Brazil.

**Keywords:** Antibodies, Transfusion reaction, Blood, Blood transfusion

## Abstract

The correct identification of erythrocyte antibodies is fundamental for the searching for compatible blood and haemolytic transfusion reactions prevention. Antibodies against antigens of high prevalence are difficult to identify because of the rarity of their occurrence and unavailability of negative red cells for confirmation. We report a case of 46-years-old woman, diagnosed with hemoglobinopathy, and who had symptomatic fall in hemoglobin levels (5.3g/dL) after blood transfusion suggestive of transfusion reaction. The patient’s blood type was O RhD-positive. Irregular antibody screening was positive and demonstrated a panreaction against all erythrocytes tested, but this result was not reactive with dithiothreitol. Using negative red cells for antigens of high prevalence of our inventory we could identify in the serum of the same erythrocytes an anti-Holley antibody associated with anti-E. Molecular analysis confirmed that the patient was negative for E and Holley antigens. The crossmath with compatible units confirmed the results. Holley is a high prevalence antigen of the Dombrock blood system whose negative phenotype is extremely rare in all populations and is associated with hemolytic transfusion reactions. This is an antibody that is difficult to identify because laboratories need to have experience in solving complex cases, and have available a large stock of rare sera and erythrocytes, as well other tools such as enzymes, thiol reagents and molecular tests. The correct identification of a rare antibody is initial and mandatory for searching of compatible donors, and to guarantee a satisfactory transfusional support.

## INTRODUCTION

The presence of alloantibodies against red blood cell (RBC) antigens in a patient serum may lead to hemolytic transfusion reactions (HTR).^1^ The correct identification of these antibodies is fundamental for the searching for compatible blood and HTR prevention. Antibodies against high prevalence antigens are difficult to identify due to the rarity of their occurrence and the unavailability of negative RBC for their confirmation. When additional compatibility issues arise besides ABO and RhD antigens, the skills and resources required for their identification are beyond the capacity of smaller and also large hospital transfusion services.^2^

The Immunohematology Reference Laboratory (IRL) of the *Hospital Israelita Albert Einstein* (HIAE) have highly complex tools, uses special techniques and have a large inventory of rare sera and RBC for identification of most RBC clinical antibodies. The IRL of HIAE has operated since the service accreditation by the American Association of Blood Banks (AABB) and has evaluated complex immunohematological cases. We report a case of a patient with a rare and clinically significant antibody against a high prevalence antigen that was identified in our IRL service.

## CASE REPORT

A 46-year-old Afro-Brazilian woman diagnosed with hemoglobinopathy, who were treated with furosemide 40mg, omeprazole 20mg, levothyroxine 75mg, complex B, folic acid and hydroxy urea. As soon the hydroxy urea was discontinued due to side effects (leg ulcers), we observed a symptomatic drop in hemoglobin levels (8.3 to 7.3g/dL). Subsequently, a RBC transfusion was requested.

After transfusion of the first RBC unit the patient presented a respiratory failure, hemodynamic instability, drop in hemoglobin levels (5.3g/dL) suggestive of an HTR. The patient was admitted to intensive care unit and remained hospitalized for several days. During hospitalization, her serum was matched with several RBC units, but all were incompatible. After recovery, the patient was discharged, although she still needed blood transfusion. At this point, the hospital sent the sample to our IRL requesting a more complex analysis for future transfusions.

Serologic testing included ABO/RhD typing, antibody screen, RBC panel, direct human antiglobulin test (DAT), and antihuman globulin (AHG) cross-match in gel test (Grifols, Spain). Antibody identification was performed using commercial panels of 11 cells previously phenotype for the main erythrocyte antigens (Bio-Rad, Brazil; Grifols, Spain) using LISS, papain and dithiothreitol (DTT). Molecular testing was performed by using the BeadChip Array assay technology (BioArray Solutions, Immucor, USA).

## RESULTS

The patient’s blood type was group O RhD positive. Direct human antiglobulin test was negative and the antibody screen was positive showing a pan reactive activity with all RBC test suggestive of an antibody against a high prevalence antigen with a title of 32. This antibody was reactive in indirect antiglobulin test (IAT) and in papain but non-reactive with DTT. Using selected RBC nonreactive with DTT and negative for high prevalence antigens from our inventory we could identify in the patient’s serum, an anti- Holley (Hy), an antibody directed to a high prevalence antigen of the Dombrock system associated with an anti-E, an antibody directed to a common antigen of the Rh system. Results of molecular analysis confirmed that the patient was negative for E and Hy antigens, and the crossmatch was negative with a blood unit phenotyped as Hy and E negative.

## DISCUSSION

Holley is a high prevalence antigen of the Dombrock blood group system (ISBT 014).^3^ The Hy-negative phenotype is extremely rare, and this phenotype is only observed among black individuals.^3 , 4^ For this reason, anti-Hy is a rare antibody and difficult to identify but associated with HTR^5^ and hemolytic disease of the fetus and newborn (HDFN).^4^ This antibody molecular basis is well defined, with the change of a single nucleotide polymorphism (323G>T) responsible for the absence of the antigen expression on RBC (HY1>HY2).^6^ Anti-Hy is difficult to identify and to do so laboratories need to have experience in solve complex cases, a large inventory of rare sera and RBC and other tools as enzymes, thiol reagents, and molecular assays.

Red blood cell and serum samples from a patient who had an hemolytic transfusion reaction was sent to our reference laboratory to identify the antibody responsible for an hemolytic transfusion reaction after transfusion of one RBC unit. As the IRL of the HIAE has all the skills and tools necessary to solve complex cases, we could identify the presence of an anti-Hy and anti-E in our patient serum. This case illustrates an example of an antibody to high prevalence antigen responsible for a severe immediate hemolytic transfusion reaction. Fortunately, a compatible donor with the patient’s phenotype was found in the state of Minas Gerais and she received a compatible blood unit with a good survival of the transfused RBC. This rare phenotype (Hy-negative) is predominant among Africans descents with a frequency of 2.4% compared with other populations.^7^

The correct identification of a rare antibody is initial and mandatory for searching compatible donors to guarantee a satisfactory transfusion support. The lack of compatible units for these patients can expose them to a potential risk of the HTR that can be avoided when properly acknowledged and addressed.
