# Supplementary material for: The rare holley antibody associated with a severe hemolytic transfusion reaction: the importance of this antibody identification to find a compatible blood unit
Source: Einstein (Sao Paulo). 2019 Sep 11;18:eRC4582. doi: 10.31744/einstein_journal/2020RC4582 (PMC6905162; doi:10.31744/einstein_journal/2020RC4582)
Supplement: Translation

# Translation: Raro anticorpo associado à reação transfusional hemolítica grave:a importância de sua identificação para encontrar uma unidade sanguínea compatível

**Authors:** Leandro Dinalli Santos, Carolina Bonet Bub, Maria Giselda Aravechia, Eduardo Peres Bastos, Jose Mauro Kutner, Lilian Castilho

**Affiliations:** 1 Hospital Israelita Albert Einstein, São Paulo, SP, Brasil.; 2 Universidade Estadual de Campinas, Campinas, SP, Brasil.

**Keywords:** Anticorpos, Reação transfusional, Sangue, Transfusão de sangue

## Abstract

A correta identificação dos anticorpos eritrocitários é fundamental na busca de sangue compatível e na prevenção das reações transfusionais hemolíticas. Anticorpos contra antígenos de alta prevalência são de difícil identificação, devido à raridade de sua ocorrência e à indisponibilidade de hemácias negativas para sua confirmação. Apresentamos aqui o caso de uma paciente do sexo feminino, 46 anos, com diagnóstico de hemoglobinopatia, que apresentou queda sintomática dos níveis de hemoglobina (5,3g/dL) após transfusão sanguínea, sugestiva de reação transfusional. O tipo sanguíneo da paciente era O RhD-positivo. A pesquisa de anticorpos irregulares foi positiva, demonstrando panreação contra todos os eritrócitos testados, mas não reativo ao ditiotreitol. Utilizando hemácias selecionadas negativas para antígenos de alta prevalência do nosso inventário, foi possível identificar no soro da mesma um anticorpo anti-Holley associado a um anti-E. A análise molecular confirmou que a paciente era negativa para os antígenos E e Holley, e as provas de compatibilidade com unidades fenotipadas confirmaram os resultados. Holley é um antígeno de alta prevalência do sistema sanguíneo Dombrock, cujo fenótipo negativo é extremamente raro em todas as populações e está associado a reações transfusionais hemolíticas. Trata-se de anticorpo de difícil identificação, pois os laboratórios precisam ter experiência na resolução de casos complexos, grande estoque de soros e eritrócitos raros, além de outras ferramentas, como enzimas, reagentes tiol e testes moleculares. A identificação correta de um anticorpo raro é inicial e obrigatória para a busca de doadores compatíveis, garantindo um suporte transfusional satisfatório.

## INTRODUÇÃO

A presença de anticorpos antieritrocitários no soro do paciente pode levar a reações transfusionais hemolíticas (RTH).^1^ A identificação correta desses anticorpos é fundamental para a busca de sangue compatível e a prevenção de RTH. Anticorpos contra antígenos de alta prevalência são difíceis de identificar por sua ocorrência rara e pela indisponibilidade de hemácias negativas para confirmação. Quando surgem problemas adicionais de compatibilidade, além dos antígenos ABO e RhD, as habilidades e os recursos necessários para identificação estão além da capacidade dos serviços de transfusão de grande e pequeno porte.^2^

O Laboratório de Referência em Imuno-hematologia (LRI) do Hospital Israelita Albert Einstein (HIAE) possui ferramentas complexas, utiliza técnicas especiais e dispõe de uma grande quantidade de soros e hemácias raras para identificação da maioria dos anticorpos clínicos eritrocitários. O LRI do HIAE opera desde seu credenciamento pela *American Association of Blood Banks* (AABB) e tem avaliado casos imuno-hematológicos complexos.

Relatamos um caso identificado em nosso LRI de uma paciente com anticorpo raro e clinicamente significativo contra antígeno de alta prevalência.

## RELATO DE CASO

Paciente do sexo feminino, negra, 46 anos, diagnosticada com hemoglobinopatia, que foi tratada com furosemida 40mg, omeprazol 20mg, levotriroxina 75mg, complexo B, ácido fólico e ureia hidroxila. Assim que a ureia hidroxila foi descontinuada devido aos efeitos adversos (úlcera de membro inferior), observou-se queda sintomática nos níveis de hemoglobina (de 8,3 para 7,3g/dL). Subsequentemente, foi solicitada transfusão de concentrado de hemácias (CH).

Após a transfusão da primeira unidade de hemácias, a paciente apresentou falha respiratória, instabilidade hemodinâmica e queda nos níveis de hemoglobina (5,3g/dL) sugestiva de RTH. A paciente foi admitida na unidade de terapia intensiva e permaneceu hospitalizada por diversos dias. Durante a internação, seu soro foi cruzado contra diversos CH, porém todos foram incompatíveis. Após recuperação, a paciente recebeu alta, apesar de ainda requerer transfusão sanguínea. Neste momento o hospital enviou amostra para o LRI solicitando análise complexa para transfusões futuras.

Os testes sorológicos incluíram tipagem ABO/RhD, rastreio de anticorpos, painel de hemácias, teste direto de antiglobulina humana (TDA) e provas de compatibilidade no teste de gel (Grifols, Espanha). A identificação dos anticorpos foi realizada por meio de painéis comerciais de 11 células previamente fenotípicas para os principais antígenos eritrocitários (Bio-Rad, Brasil; Grifols, Espanha) utilizando LISS, papaína e ditiotreitol (DTT). O teste molecular foi realizado usando a tecnologia de ensaio *BeadChip Array* (BioArray Solutions, Immucor, Norcross, GA, EUA).

## RESULTADOS

O tipo sanguíneo da paciente foi classificado como O RhD positivo. O teste direto de antiglobulina humana apresentou resultado negativo e rastreio de anticorpos foi positivo, demonstrando reatividade contra todos os eritrócitos sugestivos de anticorpo contra antígeno de alta prevalência com título de 32. Esse anticorpo foi reativo no teste de antiglobulina indireto e na papaína, mas não foi reativo com o DTT. Por meio do uso de hemácias selecionadas não reativas com DTT e negativas para antígenos de alta prevalência do nosso inventário, foi possível identificar no soro da paciente um anti-Holley (Hy), anticorpo dirigido a antígeno de alta prevalência do sistema Dombrock associado a um anti-E, anticorpo dirigido a um antígeno comum do sistema Rh. Os resultados da análise molecular confirmaram que a paciente era negativa para os antígenos E e Hy e provas de compatibilidade foram negativas contra unidades fenotipadas Hy e E negativas.

## DISCUSSÃO

Holley é um antígeno de alta prevalência do sistema do grupo sanguíneo Dombrock (ISBT 014).^3^ O fenótipo HY-negativo é extremamente raro, e este fenótipo é observado apenas entre indivíduos negros.^3 , 4^ Por essa razão, o anti-Hy é raro e de difícil identificação, mas é associado à RTH^5^ e à doença hemolítica do feto e do recém-nascido (HDFN - *hemolytic disease of the fetus and newborn* ).^4^ Esta base molecular de anticorpo é bem definida, com a mudança de um polimorfismo de nucleotídeo único (323G>T) responsável pela ausência da expressão de antígeno nas hemácias (HY1>HY2).^6^ É difícil identificar o Anti-Hy e, para isso, os laboratórios precisam ter experiência na solução de casos complexos, uma grande quantidade de soros e eritrócitos raros, e outras ferramentas, como enzimas, reagentes tiol e ensaios moleculares.

Amostras de eritrócitos e soro de uma paciente que teve reação transfusional hemolítica foram enviadas ao nosso laboratório de referência, para identificar o anticorpo responsável pela reação transfusional hemolítica após a transfusão de unidade de hemácias. Como o LRI do HIAE possui habilidades e ferramentas necessárias para resolução casos complexos, foi possível identificar a presença de um anti-Hy e anti-E no soro da paciente. Neste relato de caso, ilustra-se um exemplo de anticorpo para o antígeno de alta prevalência, responsável por uma reação transfusional hemolítica grave e imediata. Felizmente, um doador compatível com o fenótipo da paciente foi encontrado no Estado de Minas Gerais, e a mesma recebeu unidade de sangue compatível, apresentando boa sobrevida das hemácias transfundidas. Este fenótipo raro (Hy-negativo) é predominante entre os descendentes africanos, com frequência de 2,4%, comparada com outras populações.^7^

A identificação correta deste anticorpo raro é inicial e obrigatória para a busca de doadores compatíveis, garantindo um suporte transfusional satisfatório. A falta de unidades compatíveis para esses pacientes pode expô-los a risco potencial de RTH, que pode ser evitado quando devidamente identificado e abordado.
